# Clinical outcome following acute ischaemic stroke relates to both activation and autoregulatory inhibition of cytokine production

**DOI:** 10.1186/1471-2377-7-5

**Published:** 2007-02-28

**Authors:** Hedley CA Emsley, Craig J Smith, Carole M Gavin, Rachel F Georgiou, Andy Vail, Elisa M Barberan, Karen Illingworth, Sylvia Scarth, Vijitha Wickramasinghe, Margaret E Hoadley, Nancy J Rothwell, Pippa J Tyrrell, Stephen J Hopkins

**Affiliations:** 1 Division of Neuroscience, The University of Liverpool, The Walton Centre for Neurology & Neurosurgery, Lower Lane, Liverpool L9 7LJ, UK; 2 Division of Medicine and Neuroscience, The University of Manchester, Hope Hospital, Salford, UK; 3 Department of Emergency Medicine, Hope Hospital, Salford, UK; 4 Biostatistics Group, The University of Manchester, Hope Hospital, Salford, UK; 5 Stroke Medicine (Neurosciences), Hope Hospital, Salford, UK; 6 Injury Research, Hope Hospital, Salford, UK; 7 Faculty of Life Sciences, The University of Manchester, UK

## Abstract

**Background:**

As critical mediators of local and systemic inflammatory responses, cytokines are produced in the brain following ischaemic stroke. Some have been detected in the circulation of stroke patients, but their role and source is unclear. Focusing primarily on interleukin(IL)-1-related mechanisms, we serially measured plasma inflammatory markers, and the production of cytokines by whole blood, from 36 patients recruited within 12 h and followed up to 1 year after acute ischaemic stroke (AIS).

**Results:**

Admission plasma IL-1 receptor antagonist (IL-1ra) concentration was elevated, relative to age-, sex-, and atherosclerosis-matched controls. IL-1β, soluble IL-1 receptor type II, tumour necrosis factor (TNF)-α, TNF-RII, IL-10 and leptin concentrations did not significantly differ from controls, but peak soluble TNF receptor type I (sTNF-RI) in the first week correlated strongly with computed tomography infarct volume at 5–7 days, mRS and BI at 3 and 12 months. Neopterin was raised in patients at 5–7 d, relative to controls, and in subjects with significant atherosclerosis. Spontaneous IL-1β, TNF-α and IL-6 gene and protein expression by blood cells was minimal, and induction of these cytokines by lipopolysaccharide (LPS) was significantly lower in patients than in controls during the first week. Minimum LPS-induced cytokine production correlated strongly with mRS and BI, and also with plasma cortisol.

**Conclusion:**

Absence of spontaneous whole blood gene activation or cytokine production suggests that peripheral blood cells are not the source of cytokines measured in plasma after AIS. Increased plasma IL-1ra within 12 h of AIS onset, the relationship between sTNF-RI and stroke severity, and suppressed cytokine induction suggests early activation of endogenous immunosuppressive mechanisms after AIS.

## Background

Accumulating evidence supports a role for inflammation in the development of, and responses to, cerebral ischaemia. Cytokines such as interleukin(IL)-1, tumour necrosis factor-α (TNF-α) and IL-6 appear to be crucial mediators of such responses [1,2], yet much remains unknown about their complex interactions in the setting of clinical stroke.

Although cytokines are detected in the brain and cerebrospinal fluid (CSF) of patients with acute ischaemic stroke (AIS) [3-5], the relevance of measurements in the peripheral circulation is uncertain. In a recent prospective study of ischaemic stroke patients, recruited early (within 12 h) after onset of symptoms, we reported early induction of established markers of inflammation, including interleukin-6 (IL-6), and strong relationships between these and both stroke severity and outcome [6,7]. Despite evidence that IL-1 is an important mediator in stroke, it has generally not been detected [3,8], other than in one small study where a very modest elevation was noted in comparison to healthy subjects [9]. However, elevated interleukin-1 receptor antagonist (IL-1ra) concentration has been reported [10,11], and elevations have been observed in circulating TNF-α [12-14] and IL-10 [15,16].

It seems reasonable to propose that the increased plasma cytokines, detected following AIS, originate from inflammatory tissue in the central nervous system (CNS), but this is difficult to prove formally, and it is possible that they originate peripherally, perhaps from leukocytes that are activated to enter the circulation in increased numbers. Increased IL-1β messenger ribonucleic acid (mRNA) expression in blood cells, following AIS has been correlated moderately with the degree of neurological impairment [17], although other studies suggest IL-1β protein production by peripheral blood cells is no different from controls [18]. There is also evidence that release of TNF-α, IL-6 and IL-8 by blood cells in response to lipopolysaccharide (LPS) may be increased in patients following AIS, relative to controls [18,19], suggesting that leukocytes of patients with AIS may be primed to release cytokines in response to a secondary stimulus.

In view of these issues we sought to further determine the relationship between inflammation and AIS, as well as to identify whether blood leukocytes might be the source of plasma cytokines. We therefore set out primarily to examine (a) whether circulating IL-1ra is elevated and related to stroke severity or outcome, and (b) whether peripheral blood IL-1β production by peripheral blood leukocytes is induced or primed, early after AIS. We additionally report data for related cytokines and inflammatory mediators, in the same context.

## Methods

### Patients, control subjects, radiology and assessment of atherosclerosis

The study was approved by the Local Research Ethics Committee and methodology for clinical evaluation was as described previously [6]. Briefly, patients over 18 years presenting at Hope Hospital, Salford, within 12 h of symptom onset of AIS, were eligible. Patients were excluded if there was any improvement in symptoms since onset, the time of onset of symptoms could not be reliably determined, or there was evidence of active malignancy. Control subjects with no history of stroke or transient ischaemic attack, without clinically evident infection necessitating medical treatment, and without a history of cognitive impairment sufficient to interfere with daily life were matched for age (± 5 years), sex and degree of atherosclerosis. Written informed consent (or assent from a relative) was obtained for all patients and control subjects.

Computed tomography (CT) brain scans were performed on an IGE CT Pace Plus 3^rd ^generation CT scanner within 24 h of admission, in order to exclude patients with primary intracerebral haemorrhage or stroke mimic from further analysis. Ischaemic stroke patients underwent a second CT brain scan at 5 to 7 days for determination of cerebral infarct volume, using a semi-automated technique [20]. Ankle brachial pressure index (ABPI) was used to classify degree of atherosclerosis where carotid duplex data were unavailable. Patients and control subjects were divided into two groups, one without significant atherosclerosis (< 50% carotid artery stenosis bilaterally, or lowest ABPI ≥ 0.92), and one with significant atherosclerosis (≥ 50% carotid artery stenosis on at least one side, or lowest ABPI < 0.92).

### Blood and urine samples

Venous blood samples were taken from patients at baseline, the next 09:00 time point where admission was before 07:00 or after 11:00, and 24 hours after admission, plus 5 to 7 days, 3 months and 12 months at 09:00. Blood was also drawn from resting control subjects at 09:00 and at a time matched to the patient's time of admission if this was before 07:00 or after 11:00. Blood was collected into tubes containing pyrogen-free heparin, and wrapped in cool-gel packs. At 1 hour after collection, blood was centrifuged at 2000 g for 30 minutes, at 4°C. Plasma was separated, frozen and stored at -70°C until analysis. Cell pellets from 10 ml of centrifuged blood were resuspended in 5 ml saline and 1 ml volumes were centrifuged at 12000 g prior to removing the supernatant and freezing at -70°C for analysis of cytokine mRNA. Urine was collected at the same time points into foil-wrapped sterile containers, wrapped in cool-gel packs, frozen and stored at -70°C until analysis.

### Whole blood stimulation

Lipopolysaccharide (LPS) stimulation of whole blood was performed to assess the potential of peripheral blood leukocytes to produce cytokines after stroke. A separate portion of whole blood was mixed with an equal volume of Roswell Park Memorial Institute medium (RPMI; Invitrogen, Paisley, UK) or RPMI containing LPS (from *E. Coli *serotype 0128:B12, Sigma, Gillingham, UK) at 200 ng/ml and incubated in triplicate 1 ml volumes in 12-well multidishes (Nalge Nunc Int. Roskilde, Denmark). After 24 hours at 37°C in a 5% CO_2 _atmosphere the contents of each well were transferred to microcentrifuge tubes and centrifuged at 12000 g for 10 mins. The supernatant was separated, frozen and stored at -70°C until analysis.

### Enzyme-linked immunosorbent assays

Plasma (or blood culture supernatant) IL-1β, sIL-1-RII, TNF-α, sTNF-RI, sTNF-RII, IL-10 and leptin were measured using sandwich enzyme-linked immunosorbent assays (ELISAs). Analytes were captured on 96-well microplates (Costar, Cambridge, MA) with analyte-specific monoclonal antibodies (IL-1β : cat no. MAB601, R&D Systems Europe Ltd., Abingdon, UK, sIL-1-RII: cat no. MAB663; R&D Systems, TNF-α : supplied by S. Poole, NIBSC, sTNF-RI: cat no. MAB625; R&D Systems, sTNF-RII: cat no. 58.177.08; Biosource, Nivelles, Belgium, leptin: cat no. MAB398; R&D Systems, IL-10: cat no. 58.132.08; Biosource). IL-1β was secondarily bound with a goat anti-human antibody (cat no. AF201NA; R&D Systems) followed by HRP-conjugated donkey anti-goat antibody (code number 705-035-147; Jackson ImmunoResearch Laboratories Inc., PA, USA), prior to development with orthophenylene-diamine (OPD; Sigma, Poole, UK). Other analytes were secondarily bound with biotinylated antibodies (sIL-1-RII: cat no. BAF263; R&D Systems, TNF-α : supplied by S. Poole (NIBSC, South Mimms, UK); sTNF-RI: cat no. BAF225, R&D Systems, sTNF-RII: cat no. 58.177.02; Biosource, leptin: cat no. BAM398; R&D Systems, IL-10: cat no. 58.132.02; Biosource) followed by streptavidin-HRP (cat no. 43–4323; Zymed Laboratories Inc., CA, USA, or cat no. 41.000.04; Biosource for the IL-10 and sTNF-RII assays) and developed with OPD. Working standards for IL-1β (R&D Systems, cat. no. 2010LB, TNF-α (supplied by S. Poole, NIBSC), IL-10 (Biosource, cat no. SD909) were calibrated against WHO international standards. Cortisol, IL-6 and IL-1ra were measured as described previously [6,21]. Other analytes were calibrated using commercially available materials: sIL-1-RII: cat no. 263-2R; R&D Systems; sTNF-RI: cat no. 225-B1; R&D Systems; sTNF-RII: cat no. 58.177.10; Biosource; leptin: cat no. 398-LP, R&D Systems.

Minimum assay sensitivities, allowing for sample dilution (1:10 for sIL-RII, sTNF-RI, sTNF-RII and leptin, and 1:5 for IL-1ra), were 6.5 pg/ml (IL-1β), 352 pg/ml (IL-1ra), 860 pg/ml (sIL-1-RII), 7 pg/ml (TNF-α), 200 pg/ml (sTNF-RI), 1000 pg/ml (sTNF-RII), < 500 pg/ml(leptin), 0.6 pg/ml (IL-10). Inter-assay coefficients of variation (CVs) for quality control samples were 15% at 83.5 pg/ml to 26% at 51.5 pg/ml for IL-1β, 11% at 2.48 ng/ml to 19% at 680 pg/ml for IL-1ra, 12% at 1.2 ng/ml to 20% at 145 pg/ml for sIL-1-RII, 11% at 42 pg/ml to 10 pg/ml for TNF-α), 7% at 200 pg/ml to 36% at 23 pg/ml for sTNF-RI, 7% at 850 pg/ml to 9% at 300 pg/ml for sTNF-RII), 22% at 67.5 ng/ml to 28% at 5.4 ng/mlfor leptin, 4% at 235.1 pg/ml to 6% at 8.2 pg/ml for IL-10. For measurements of supernatants from unstimulated samples, minimum sensitivities were 6.7 pg/ml (IL-1β), 3.4 pg/ml (TNF-α) and 7 pg/ml (IL-6) and interassay CVs were 7% at 56.3 pg/ml to 30% at 5.9 pg/ml for IL-1β, 11% at 99.5 pg/ml to 19% at 14.5 pg/ml for TNF-α, and 11% at 45 pg/ml, 23% to 45% at 5.3 pg/ml for IL-6. For measurements of supernatants from LPS-stimulated samples, minimum sensitivities were 7% for IL-1β, 8.5 pg/ml for TNF-α, 7 pg/ml for IL-6, and 1.6 pg/ml for IL-10. Interassay CVs were 6% at 911 pg/ml to 12% at 19 pg/ml for IL-1β, 10% at 4.6 ng/ml to 19% at 14 pg/ml for TNF-α, 7% at 1.3 ng/ml to 23% at 16.9 pg/ml for IL-6, and 10% at 261 pg/ml to 16% at 12 pg/ml for IL-10. Cytokine production was corrected for monocyte counts obtained at each time point, and adjusted for the 1:1 dilution with RPMI.

### Neopterin and creatinine measurement

The urinary neopterin/creatinine ratio was determined by reverse-phase high performance liquid chromatography (Spectroflow 400 isocratic pump; Applied Biosystems, Warrington, UK) at ambient temperature using a 4.6 × 100 mm analytical column packed with 3 μm particles of Spherisorb ODS2 (Waters, Milford, MA), preceded by a 4.6 × 10 mm guard cartridge packed with a generic C18 material. Elution was with a gradient of 15 mmol/l potassium phosphate buffer, pH 6.4, at 0.8 ml/min for 6 min followed by the addition of 6% acetonitrile at 0.8 ml/min for a further 3 minutes. Neopterin was measured fluorimetrically (excitation 353 nm, emission 438 nm: LS-5 luminescence spectrophotometer; Perkin-Elmer, Beaconsfield, UK) and the ratio was calculated relative to creatinine determined by ultraviolet absorbance at 235 nm (Spectroflow 757 absorbance detector; Applied Biosystems). Urine samples were diluted 1/10 in 15 mmol/l potassium phosphate buffer, pH 6.4, containing 0.2% disodium EDTA, prior to injection of 20 μl on to the column. Urinary neopterin was expressed as a ratio with creatinine to account for variation in urine volumes. Inter-assay coefficients of variation for urinary neopterin/creatinine were 11.5% at a ratio of 259, 23.4% at 178 and 19.8% at 101.

### Measurement of cytokine mRNA in whole blood

RNA was isolated from the frozen blood cell pellets of 20 sequentially admitted patients, and their matched controls, using Trizol^LS ^RNA isolation solution (Invitrogen Ltd., Paisley, UK). The RNA concentration was determined spectrophotometrically at 260 nm. All RNA samples for IL-1β mRNA measurements were routinely treated with 0.5 units RQ1 DNase (Promega, UK) per 50 ng total RNA at 37°C for 30 mins. The IL-1β, IL-6 and TNF-α mRNA in these samples were measured using an immunometric, reverse transcriptase, polymerase chain reaction, as previously described [22]. Where IL-6 and TNF-α mRNA values were high, these were also DNase treated to, although in no case were results substantially reduced by this treatment. Cytokine mRNA was expressed as molecules of mRNA/monocyte, assuming total RNA recovery from blood was 100%.

### Statistical Analysis

The pre-specified, primary outcome measures were peak plasma IL-1ra and maximum IL-1β production by whole blood during the first week after ischaemic stroke compared with CT cerebral infarct volume at 5 to 7 days and modified Rankin Scale (mRS) at 3 months. Post-hoc evaluation of the data resulted in a change in the IL-1β parameter to minimum production. Correlations were assessed using the Spearman Rank correlation coefficient. Paired *t*-tests or logistic regression analysis of log-transformed cytokine, cytokine receptor, leptin, urinary neopterin, or cytokine production data were used for group comparisons where appropriate. The sample size of 36 patient-control subject pairs had 80% power at the 5% significance level to detect a difference of 0.67 standard deviation (SD) in primary analyses between control subjects and patients. All calculations were performed using SPSS for Windows (SPSS Inc., Chicago, IL). Secondary analyses were plasma IL-1β, sIL-1RII, TNFα, sTNF-RI, sTNF-RII, IL-10, leptin and urinary neopterin, and IL-1β, IL-6 and TNF-α production (a) at all time points compared with controls, (b) peak in the first week versus CT infarct volume at 5 to 7 days and clinical outcome at 3 and 12 months. Secondary analyses are descriptive, to be used for the purpose of hypothesis generation.

## Results

Forty-three patients were recruited between April 2000 and January 2001. Six patients with primary intracerebral haemorrhage and one patient for whom no matched control could be found, were excluded. The thirty-six remaining patients included in the analysis were recruited at a median interval of 4.75 h (range 1.5 h to 11.75 h) from onset of symptoms of AIS. Fourteen patients died by 12 months: certified causes of death were index stroke (8), recurrent stroke (1), pulmonary embolism (1), left ventricular failure secondary to myocardial infarction (1) and sepsis (3). Infections and other events associated with an inflammatory response occurring in the 6-week period prior to each assessment were as described previously [6]. Participant characteristics are summarised in table 1. No patients received treatment with intravenous thrombolysis.

### Plasma cytokines and soluble receptors

**Figure 1:**
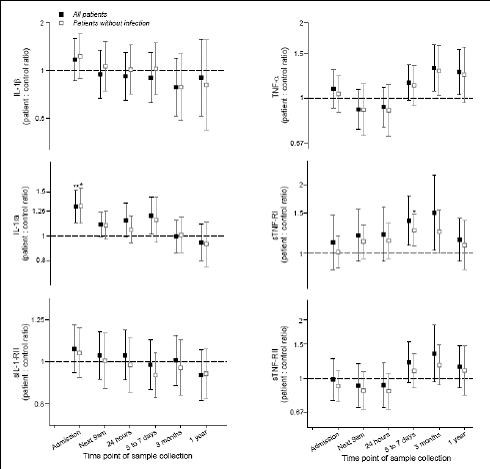
Plasma cytokine kinetics in all patients and patients without infection (in the 6 weeks preceding assessment). Patient values are expressed as multiples (ratios) of their corresponding controls (with 95% CIs) to account for skewed distributions. The 24 h time point includes "next 9 am" data where admission was between 07:00 and 11:00. *p ≤ 0.01 **p ≤ 0.001 (relative to controls).

The median plasma IL-1ra concentration was significantly elevated relative to controls at admission (p < 0.001), and remained elevated until 5–7 days (fig 1). Peak IL-1ra concentration in the first week showed modest correlation with infarct volume (r = 0.45, p = 0.02), but did not correlate significantly with modified Rankin Scale (mRS) at 3 months, or show meaningful correlations with any other outcome measures. Plasma IL-1β and sIL-1-RII concentrations were similar to controls at all time points (fig 1) and also did not exhibit meaningful correlations with outcome. Peak IL-1ra, IL-1β and sIL-1-RII concentrations did not exhibit any correlations with each other. Plasma sTNF-RI concentration was elevated at 5–7 days, in patients without evidence of infection (fig 1). Peak plasma sTNF-RI concentration in the first week correlated strongly with infarct volume (r = 0.62, p = 0.001), mRS at 3 months (r = 0.59, p < 0.001) and 1 year (r = 0.57, p = 0.001), and Barthel Index (BI) at 3 months (r = -0.58, p = 0.001) and 1 year (r = -0.61, p < 0.001). Plasma TNF-α and sTNF-RII concentrations followed a similar but less marked pattern to sTNF-RI (fig 1), and peak plasma TNF-α, sTNF-RI and sTNF-RII concentrations all showed strong correlations with each other (TNF-α :sTNF-RI, r = 0.64, p < 0.001; TNF-α : sTNF-RII, r = 0.77, p < 0.001; sTNF-RI:sTNF-RII, r = 0.83, p < 0.001). Plasma IL-10 and leptin concentrations were similar to controls at all time points (not shown).

### Peripheral blood cytokine production and gene expression

**Figure 2:**
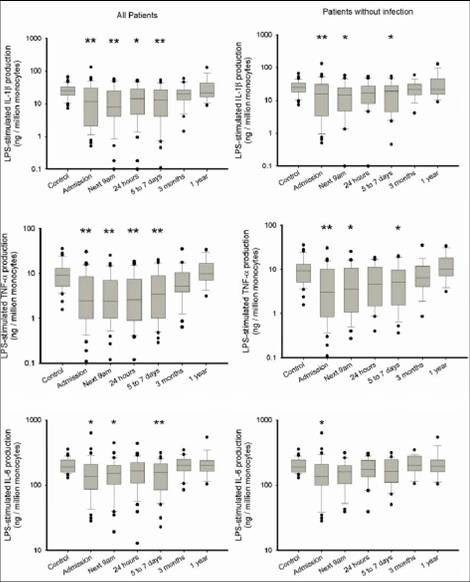
Lipopolysaccharide-stimulated cytokine production kinetics in all patients and patients without infection (in the 6 weeks preceding assessment). Box plots show medians, 10^th ^and 90^th ^percentiles and outliers. The 24 hour timepoint includes "next 9 am" data where admission was between 07:00 and 11:00. *p ≤ 0.01 **p ≤ 0.001 (relative to controls).

**Figure 3:**
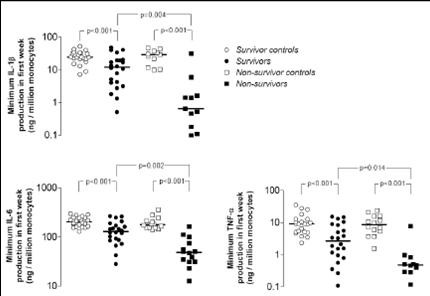
Minimum LPS-stimulated cytokine production in first week in survivors and non-survivors to 12 months. P values generated from analysis of log-transformed data (logistic regression for comparison of survivors and non-survivors, paired t-test for comparisons with controls).

Following activation with LPS, the cytokines IL-1β, IL-6 and TNF-α were all strongly induced in whole blood. An 850-fold increase was seen in IL-6 production at admission compared with blood incubated in medium. However, IL-1β production in blood from patients was significantly reduced relative to controls between admission and 5–7 days, and a similar pattern was apparent for TNF-α and IL-6 (fig 2). This pattern was seen in blood from all patients, although it was less evident in those without infection. IL-10 production, measured at admission and 24 hours after admission, exhibited a similar pattern to IL-1β, TNF-α and IL-6 production at these timepoints (not shown). Minimum *in vitro *IL-1β production in the first week correlated significantly with mRS at 3 months (table 2). A similar correlation was apparent between IL-6 production and mRS, although the correlation between TNF production and mRS was less strong (table 2). Minimum *in vitro *IL-1β production was lower in non-survivors than in survivors at 12 months (p = 0.004) (figure 3), and the same was apparent for IL-6 (p = 0.002) (figure 3) and TNF-α (p = 0.014) (figure 3). Plasma cortisol concentration showed strong inverse correlations with LPS-stimulated IL-1β, IL-6 and TNF-α production between admission and 5–7 days (table 3). In the absence of LPS, blood incubated in medium for 24 h produced minimal amounts of IL-1β (Interquartile range (IQR) for all time points of 22 to 47 pg/10^6 ^monocytes (controls), 19 to 66 pg/10^6 ^monocytes (patients)), IL-6 (IQR for all time points 28 to 55 pg/10^6 ^monocytes (controls), 28 to 102 pg/10^6 ^monocytes (patients)) or TNF-α (IQR for all time points 39 to 82 pg/10^6 ^monocytes (controls), 35 to 90 pg/10^6 ^monocytes (patients)). Minimal spontaneous activation of peripheral blood cells was confirmed by the absence of cytokine mRNA expression in a group of 20 consecutive patients examined either at admission or 24 h later (data not shown).

### Urinary neopterin

Urinary neopterin was raised in patients at 5–7 days (median 354 μmol/mol creatinine) compared to controls (median 254 μmol/mol creatinine) (p = 0.002), but not at any other time point and a similar elevation was apparent in patients with evidence of infection excluded. Peak urinary neopterin correlated strongly with peak TNF-α concentration (r = 0.6, p < 0.001), but less with sTNF-RI (r = 0.4, p = 0.05) and sTNF-RII (r = 0.4, p = 0.01), and appeared unrelated to other plasma cytokines, soluble receptors or *in vitro *cytokine production.

### Atherosclerosis

Seventeen patients and control subjects were classified as having significant atherosclerosis. A higher urinary neopterin concentration was seen in control subjects with significant atherosclerosis (median 309 μmol/mol creatinine) than those without (median 244 μmol/mol creatinine) (p < 0.01). IL-10 concentration was lower in control subjects with significant atherosclerosis (median 0.60 pg/ml) than those without (median 1.00 pg/ml) (p = 0.05). No notable differences in other plasma cytokines, soluble receptors or *in vitro *cytokine production were seen between those individuals (including patients and controls) with or without significant atherosclerosis at any time-point.

## Discussion

Cytokines such as IL-1, TNF and IL-6 are key regulators of inflammation at sites of infection or tissue damage. Following AIS, it seems reasonable to hypothesise that cytokines measured in the plasma arise from cells associated with inflammatory activity in the brain, but this is not certain. Therefore, in addition to evaluating the relationships between plasma cytokines and the clinical impact of stroke, we have attempted to identify the possible contribution of peripheral blood leukocytes to the plasma cytokine pool. We also wanted to know whether leukocyte activation, apparent in terms of increased numbers in the circulation [6], was associated with an increased potential to produce cytokines. In this study our primary focus has been the IL-1 system, but we also examined other cytokines, their receptors, and neopterin, as molecules that have previously been associated with inflammatory cell activation.

In accord with its localised role, and with previous studies [3,8], there was no evidence of an increase in plasma IL-1β concentration. We also found no change in plasma sIL-1-RII concentration. However, the elevation in plasma IL-1ra concentration, at a much earlier stage after stroke onset than previously reported [10], suggests early activation of anti-inflammatory components directed at regulating IL-1. Leptin was evaluated because it appears in some cases to mediate neuroimmune responses dependent on IL-1 [23] and, although high leptin levels were recently found to be associated with stroke [24], the present data support neither a clear change in leptin concentration following stroke, nor any relationship with outcome.

Our findings with respect to TNF-α are consistent with one previous study [8], but differ from those showing that plasma or serum TNF-α concentrations after stroke are increased and correlate with cerebral infarct volume [12-14]. The role of TNF-α in stroke remains controversial, and indeed a potentially beneficial role in ischaemic tolerance has been suggested [25]. However, we found sTNF-RI was increased at 5–7 d and the peak concentration in the first week correlated with infarct volume and outcome (mRS and BI) at 3 months and 1 year. This is consistent with earlier observations of elevated sTNF-RI concentration in stroke patients with nosocomial infection [26]. A more robust relationship between sTNF-RI and stroke severity or outcome may reflect a longer half-life of receptors compared to TNF-α. The possibility that TNF-α and its receptors are activated in concert is supported by the strong correlations between their peak concentrations and suggests their release is proportionate to each other. The fact that we used controls matched for atherosclerosis, and that elevated serum sTNF-RI and sTNF-RII concentrations are increased in carotid atherosclerosis [27] may explain why our data are not as clearly differentiated in respect of TNF-α as those where healthy controls were used.

Atherosclerosis may also be a confounding factor with IL-10 analysis, since patient plasma IL-10 concentrations were similar to those of controls and reduced concentrations of plasma IL-10 have been noted in studies where plasma of patients with stroke has been compared to that of healthy controls [15]. In support of this explanation, higher plasma concentrations of IL-10 have been detected in plasma of patients with stable, versus unstable coronary syndromes [28] and, experimentally, IL-10 deficiency results in increased atherosclerosis in apolipoprotein E-deficient mice [29].

Activation of cells of monocytoid lineage is associated with increased production of neopterin in plasma or urine. Although we found urinary neopterin to be increased following stroke, and this is consistent with a previous report that found neopterin to be increased during the first week after stroke [30] we also found it to be higher in control subjects with significant atherosclerosis. The association with atherosclerosis has been reported previously [31] and it is possible that this influences the strength of the apparent association. Consistent with this interpretation, another study reported neopterin as being higher one year after stroke than at the acute stage, and to be correlated with age [32].

CSF is arguably more relevant than plasma to events occurring in the brain. However, lumbar puncture poses a risk of cerebral herniation and it is difficult to obtain CSF at repeated time points. Plasma, or serum markers therefore have potentially greater practical value and have been evaluated in most studies. It may be that plasma cytokines do reflect brain cytokines, particularly after an acute cerebral event, but the validity of this has not been well established, and blood monocytes in particular are potential sources of plasma cytokines. This is particularly the case where serum, rather than plasma, has been collected and there is potential for monocytes to release cytokines during clotting, particularly if endotoxin is present in collection tubes [33,34]. The ability of blood cells to produce cytokines is potentially relevant, not only because they may be responsible for producing the cytokines while in the circulation but because these cells have the potential to enter the CNS after stroke and their activation state may reflect systemic responsiveness of the immune system after stroke. Our determination of cytokine gene activation in the blood of patients, and of spontaneous, whole blood cytokine induction, indicates peripheral blood cells are not the source of cytokines after ischaemic stroke. This supports the hypothesis that cytokine production is upregulated within the brain, possibly by microglia, in response to cerebral ischaemia, and these cytokines enter the peripheral circulation via reabsorption from the CSF, or via afferent lymphatics. While cells in other peripheral tissues, such as those associated with concomitant peripheral atherosclerosis, might also be expected to be contributors to a pre-existing inflammatory state, it seems unlikely that they would be more readily activated than blood cells following a stroke.

In contrast to the increased plasma cytokines, presumed to originate from the brain, we report, for the first time, reduction of cytokine production by endotoxin-stimulated whole blood from patients relative to controls. This was seen within hours of ischaemic stroke and persisted during the first week. These data differ from a previous report, where LPS-induced more IL-6 and TNF in blood from patients with stroke, compared to controls [19]. However, they are consistent with a number of studies examining cytokine production by whole blood from trauma patients [35,36]. Reasons for conflict with the earlier study [19] are not clear, although in that study: (1) cytokines were induced for only 4 h by LPS, which might be relevant if downregulation is relatively delayed; (2) data were not corrected for monocyte count; (3) non-normally distributed data were analysed with parametric statistical tests and (4) controls were not matched for degree of atherosclerosis. Suppression of endotoxin-stimulated cytokine production, and the inverse relationships with stroke severity and outcome, may relate to the extent of CNS inflammation and a physiological attempt to downregulate cytokine activation, although it should be noted that the correlation with infarct volume was relatively weak. The mechanisms are therefore unclear although, following trauma, a suppressive factor has been shown to be present in serum and this was apparently not transforming growth factor (TGF)-β, IL-4 or IL-10 [36]. IL-10 seems unlikely to have contributed to the relative reduction in cytokine production observed in the present study because its production pattern was similar to those of IL-1β, IL-6 and TNF-α. It seems unlikely that reduced capacity to produce cytokines is due to depletion in vivo, since there was no evidence for increased IL-1β or TNF-α in the plasma, and mRNA for all three cytokines had not been induced at baseline.

We have previously reported elevation in plasma cortisol concentration within 12 hours of ischaemic stroke [6] and the correlation between plasma cortisol and suppression of cytokine production, suggests this might be a potential explanation. These observations are consistent with experimental data describing a systemic anti-inflammatory response, including reduced endotoxin stimulated whole blood TNF-α and increased IL-10 production, after infusion of IL-1β into the brain, through stimulation of the hypothalamo-pituitary-adrenal axis and the sympathetic nervous system [37]. However, evidence from experimental ischaemia suggests that activation of the sympathetic nervous system, but not corticosteroid receptors, is more important in suppression of whole blood TNF production [38]. Both catecholamines and corticosteroids suppress endotoxin-induced cytokine production by whole blood from healthy volunteers [39,40]. Such regulatory responses, and increases in inhibitory cytokines such as IL-1ra, may be responsible for increased vulnerability of stroke patients to infections [38,41,42]. This might contribute toward the increased mortality in patients with greatest inhibition of systemic cytokine induction.

Particular strengths of the present study are the very early recruitment of patients after onset of symptoms of AIS (median < 5 h), and further assessments within the first 24 h of admission. In studies where later sampling time points have been used it is difficult to distinguish the effects of the stroke itself from subsequent infections or other events. We also recognise that cytokine responses seen at 3 months and 1 year reflect the fact that many patients with the most severe strokes did not survive to later time points. Control subjects were individually matched for degree of atherosclerosis, in addition to age and sex, because each of these variables are known to influence peripheral inflammatory markers, including IL-6 and neopterin [43]. The rationale for carotid artery stenosis and ABPI thresholds for 'significant atherosclerosis' used in the present study was guided by the available evidence, as discussed previously [6]. Possible criticisms of the present study might include our relatively small sample size and multiple statistical testing. We may have missed some associations through the former, and may have identified spurious associations through the latter. However, we clearly stated the primary questions at the study outset and believe that we are reporting data that should provide an important stimulus for further study.

## Conclusion

The present data confirm the association between peripheral inflammatory markers and stroke and also draw attention to the potential confounding issue of the association between stroke and atherosclerosis. Peripheral blood cells appear to make no major contribution to the plasma cytokine concentration after stroke and, compared to age-, sex-, and atherosclerosis-matched control subjects, are relatively resistant to activation by LPS. This reduced production of cytokines is significantly correlated with worse outcome and mortality, and highlights the potential of identifying vulnerable subjects for treatment.

## Competing interests

The author(s) declare that they have no competing interests.

## Authors' contributions

HCAE conducted the study and analysed the data, carried out assays and drafted the manuscript. CJS, CMG, RFG and EMB all participated in the conduct of the study including patient recruitment, follow-up assessments and sample preparation. KI, SS, VW and MEH carried out assays and cytokine mRNA measurements. AV advised on study design and statistical analysis. NJR, PJT and SJH conceived of the study, secured funding, and participated in its design and coordination. All authors read and approved the final manuscript.

## Pre-publication history

The pre-publication history for this paper can be accessed here:



## Figures and Tables

**Table 1:** Control subject and patient characteristics

*Baseline characteristics*	Controls	Patients		
Mean age ± S.D. (years)	68.7 ± 12.6	69.6 ± 13.0		
Male	24 (67%)	24 (67%)	*Stroke characteristics*	
			
Caucasian	36 (100%)	36 (100%)	*Oxfordshire Community Stroke Project (OCSP) classification*	
Systolic blood pressure*	142 (100, 200)	151 (60, 232)	Total anterior circulation syndrome	16 (46%)
Diastolic blood pressure*	79 (60, 120)	82 (40, 146)	Partial anterior circulation syndrome	12 (34%)
Body mass index* (kg/m^2^)	26 (17, 36)	26 (18,41)	Lacunar syndrome	5 (14%)
'Significant' atherosclerosis	17 (47%)	17 (47%)	Posterior circulation syndrome	2 (6%)
			
Current or former smoker	23 (64%)	25 (69%)	National Institutes of Health Stroke Scale (NIHSS) score*	
Smoking history unknown	0 (0%)	1 (3%)	Admission	14 (3, 24)
Hypertension	11 (31%)	23 (64%)	5–7 days	11 (1, 42)
			
Atrial fibrillation	2 (6%)	8 (22%)	Modified Rankin Scale*	
Diabetes mellitus	6 (17%)	3 (8%)	Pre-stroke	0 (0, 4)
			
Ischaemic heart disease	7 (19%)	8 (22%)	Barthel Index*	
Prevalence of selected drugs**			Pre-stroke	100 (20, 100)
			
Paracetamol	Not known	20 (56%)		
Statins	11 (31%)	20 (56%)		
Antiplatelet agents	14 (39%)	33 (92%)	*Infections/other events associated with an inflammatory response*||	
			
Warfarin	2 (6%)	4 (11%)	Pre-stroke	3 (8%)¶
ACEIs† or A-IIR‡ antagonists	5 (14%)	10 (28%)	Pre-stroke or 1^st ^week after stroke	11 (33%)¶
NSAIDs§	4 (11%)	7 (19%)		
Beta-blockers	7 (19%)	15 (42%)	Pre-3 months	5 (21%)¶
Inhaled corticosteroids	2 (6%)	6 (17%)	Pre-1 year	3 (17%)¶
Azathioprine	1 (3%)	0 (0%)		

* data shown as median (min, max)

** at recruitment for control subjects; during study period for patients

† angiotensin-converting enzyme inhibitors

‡ angiotensin-II receptor

§non-steroidal anti-inflammatory drugs

|| in 6 weeks prior to assessment

¶number of patients (% of survivors) with infection at each time point

**Table 2:** Correlation matrix of cytokine production versus stroke severity and outcome

	Minimum cytokine production in first week					
	
	IL-1β		IL-6		TNF-α	
	
	r	p	r	p	r	p
Admission NIHSS	-0.45	0.008	-0.48	0.003	-0.36	0.04
5–7 day NIHSS	-0.34	0.05	-0.30	0.08	-0.27	0.1
Infarct volume	-0.43	0.03	-0.37	0.06	-0.31	0.1
3 month mRS	-0.63	< 0.001	-0.58	< 0.001	-0.48	0.005
1 year mRS	-0.65	< 0.001	-0.65	< 0.001	-0.55	0.002
3 month BI	0.59	< 0.001	0.57	< 0.001	0.47	0.008
1 year BI	0.62	< 0.001	0.65	< 0.001	0.55	0.002

r values are Rank Spearman correlation coefficients

**Table 3:** Correlation matrix of plasma cortisol concentration versus LPS-stimulated whole blood in vitro cytokine production

		IL-1β production		IL-6 production		TNF production	
		
		r	p	r	p	r	p
Control subjects	09:00	-0.09	0.6	-0.001	1.0	-0.39	0.02
	Admission matched†	0.18	0.3	-0.02	0.9	-0.06	0.7

Patients	Admission	-0.57	< 0.001	-0.59	< 0.001	-0.72	< 0.001
	24 hours	-0.65	< 0.001	-0.64	< 0.001	-0.67	< 0.001
	5–7 days	-0.58	0.001	-0.48	0.006	-0.68	< 0.001
	3 months	-0.05	0.8	-0.13	0.5	-0.10	0.6
	12 months	-0.35	0.2	-0.09	0.7	-0.17	0.5

r values are Rank Spearman correlation coefficients

†admission matched data includes 09:00 values if admission was between 07:00 and 11:00
